# Rhamnolipid biosurfactants—past, present, and future scenario of global market

**DOI:** 10.3389/fmicb.2014.00454

**Published:** 2014-09-02

**Authors:** Kamaljeet K. Sekhon Randhawa, Pattanathu K. S. M. Rahman

**Affiliations:** ^1^Section for Sustainable Biotechnology, Department of Biotechnology, Chemistry and Environmental Engineering, Aalborg UniversityCopenhagen, Denmark; ^2^Technology Futures Institute, School of Science & Engineering, Teesside UniversityTees Valley, UK

**Keywords:** biosurfactants, rhamnolipids, market Access, surfactants, sustainability

## Rhamnolipids—brief outline

Biosurfactants, widely known as surface-active agents of biological origin, have carved a niche for themselves in the market due to their unique environment-friendly properties. They have come a long way since first biosurfactant “surfactin” was purified and characterized by Arima et al. (1968). Biosurfactants have been researched thoroughly and satisfactorily since then by many research groups across the world yet there are aspects that elude our understanding. There are five major categories of biosurfactants viz. glycolipids, phospholipids and fatty acids, lipopeptides and lipoproteins, polymeric biosurfactants and particulate biosurfactants that have found applications in agricultural, pharmaceutical, food, cosmetics, and detergent industries. Data reveals there are more than 250 patents obtained on these wonder biodegradable molecules so far (Shete et al., 2006; Rahman and Gakpe, 2008). It has also been observed that microbial biosurfactants are advantageous over plant-based surfactants because of the scale-up capacity, rapid production, and multi-functional properties. Several plant-based biosurfactants for example saponins, lecithins, and soy proteins have excellent emulsification properties but are expensive to produce at industrial scale and have other debatable issues such as solubility and hydrophobicity (Xu et al., 2011).

Among the various categories of biosurfactants the glycolipid biosurfactants “rhamnolipids” stand apart. Rhamnolipid, primarily a crystalline acid, is composed of β-hydroxy fatty acid connected by the carboxyl end to a rhamnose sugar molecule. Rhamnolipids are predominantly produced by *Pseudomonas aeruginosa* and classified as: mono and di-rhamnolipids. Other *Pseudomonas* species that have been reported to produce rhamnolipids are *P. chlororaphis*, *P. plantarii, P. putida*, and *P. fluorescens*. Some bacteria are known to produce only mono-rhamnolipids while some produce both. The ratio of mono and di-rhamnolipid can also be controlled in the production method. There are enzymes available that can convert mono-rhamnolipids into di-rhamnolipids. In 1984, the first patent for the production of rhamnolipids was filed by Kaeppeli and Guerra-Santos (US 4628030) and obtained in 1986 for their work on *Pseudomonas aeruginosa* DSM 2659 (Kaeppeli and Guerra-Santos, 1986). Subsequently, Wagner et al. filed a patent (US 4814272) in 1985 for the biotechnical production of rhamnolipids from *Pseudomonas sp*. DSM 2874 and obtained the same in 1989 (Wagner et al., 1989). In the past close to three decades, there has been a great body of research work carried on rhamnolipids revealing many of their astonishing applications and making them reach the pinnacle of popularity among all the categories of biosurfactants in the global market. The reason behind the current global interest in rhamnolipid production owes to their broad range of applications in various industries along with many spectacular “eco-friendly” properties.

The current critique articulates to present opinion on rhamnolipid research and is an attempt to retrospect what brings rhamnolipids in the forefront. This article is a bird's-eye view on a timeline of rhamnolipids story so far and also a critical analysis on why despite so many patents and research work rhamnolipids still do not rule the global biosurfactant market.

## Inimitable applications of rhamnolipids

Over the years rhamnolipids are becoming broadly pertinent in various industries and are posing a serious threat to the synthetic surfactants. Before venturing into the current production economics of rhamnolipids it is imperative to evaluate the major applications of rhamnolipids that make them noticeable among other biosurfactants. A list of five major applications of rhamnolipids that cater to the wide range of industrial demands includes:

1. *Bioremediation and enhanced oil recovery (EOR)*: Rhamnolipids show excellent emulsification properties, efficiently remove crude oil from contaminated soil and facilitate bioremediation of oil spills (Rahman et al., 2003; Costa et al., 2010).
2. *Pharmaceuticals and therapeutics*: Rhamnolipids show low toxicity, surface active properties and antimicrobial activities against several microbes (*Bacillus cereus, Micrococcus luteus, Staphylococcus aureus, Listeria monocytogenes*) thereby showing promising applications in pharmaceuticals and therapeutics (Magalhaes and Nitschke, 2013).
3. *Cosmetics*: Rhamnolipid as an active ingredient is found to be effective for several skin treatments i.e., wound healing with reduced fibrosis, cure of burn shock, treatment of wrinkles hence are in demand in the health and beauty industry (Piljac and Piljac, 2007).
4. *Detergents and cleaners*: Rhamnolipids are natural emulsifiers and surface active agents leading to their wide spread usage in detergent compositions, laundry products, shampoos and soaps (Parry et al., 2013).
5. *Agriculture*: Rhamnolipids are already used for soil remediation for improving soil quality and are now further getting explored for plant pathogen elimination, for aiding the absorption of fertilizers and nutrients through roots and as biopesticides (Sachdev and Cameotra, 2013).

## Biosurfactant producing companies—with focus on rhamnolipids

Rhamnolipids are highly applicable in various activities with some researchers advancing the technology from laboratory to higher scale. However, there still are very limited companies in the field which are producing biosurfactants at a marketable scale. We tried to compile a list of biosurfactant producing companies around the globe (Table 1). The compilation evidently defines which biosurfactants are mostly researched and produced at higher scale.

**Table 1:** **Biosurfactant producing companies around the globe**.

**S. No**.	**Company**	**Location(s)**	**Product(s)**	**Focus on**
1	TeeGene Biotech	UK	Rhamnolipids and Lipopeptides	Pharmaceuticals, cosmetics, antimicrobials and anti-cancer ingredients
2	AGAE Technologies LLC	USA	Rhamnolipids (R95, an HPLC/MS grade rhamnolipid)	Pharmaceutical, cosmeceutical, cosmetics, personal care, bioremediation (*in situ* & *ex situ*), Enhanced oil recovery (EOR)
3	Jeneil Biosurfactant Co. LLC	USA	Rhamnolipids (ZONIX, a bio-fungicide and RECO, a rhamnolipid used in cleaning and recovering oil from storage tanks)	Cleaning products, EOR
4	Paradigm Biomedical Inc.	USA	Rhamnolipids	Pharmaceutical applications
5	Rhamnolipid Companies, Inc.	USA	Rhamnolipids	Agriculture, cosmetics, EOR, bioremediation, food products, pharmaceutical
6	Fraunhofer IGB	Germany	Glycolipids, Cellobiose lipids, MELs	Cleansing products, shower gels, shampoos, washing-up liquids, pharmaceutical (bioactive properties)
7	Cognis Care Chemicals	China, Germany, USA	Alkyl polyglucoside APG^®^, Plantacare 1200 GLY (green surfactant for use in oral-dental formulations), Rheocare TTA (for cleansing formulations)	Used in formulations for household cleaners, bath/shower gels, dish washing, laundry detergents and in agrochemicals
8	Saraya Co. Ltd.	Japan	Sophorolipids (Sophoron, a low-foam dishwasher detergent)	Cleaning products, hygiene products
9	Ecover Belgium	Belgium	Sophorolipids	Cleaning products, cosmetics, bioremediation, pest control, pharmaceuticals
10	Groupe Soliance	France	Sophorolipids	Cosmetics
11	MG Intobio Co. Ltd.	South Korea	Sophorolipids (Sopholine—functional soap with Sophorolipids secreted by yeasts)	Beauty and personal care, bath supplies e.g., soaps with new functions
12	Synthezyme LLC	USA	Sophorolipids	Cleaning products, cosmetics, food products, fungicides, crude oil emulsification
13	Allied Carbon Solutions (ACS) Ltd	Japan	Sophorolipids (ACS-Sophor—first bio-based surfactant from Indian mahua oil)	Agricultural products, ecological research
14	Henkel	Germany	Sophorolipids, Rhamnolipids, Mammoslyerthritol lipids	Glass cleaning products, laundry, beauty products
15	Lion Corporation	Japan	Methyl ester sulfonates (MES)	Detergents formulations, cleaning products
16	Lipo Chemicals	USA	Lipomulse Luxe (high-temperature resistance emulsifier)	Skin care, sun-lotions hair care formulations, thickening polymers, rheological modifiers, natural gums
17	Kaneka Co.	Japan	Sophorose lipids	Cosmetics and toiletry products

## Availability of feedstock and its impact on biosurfactants

Biodiesel is produced by the trans-esterification of vegetable oils and fats with methanol in the presence of a catalyst. Glycerol is received as a by-product from this reaction. The production of 1 ton biodiesel generates about 100 kg of glycerol. Hence, the European biodiesel industry might release about 600 Kiloton glycerol per year with an increasing tendency in Europe and worldwide. Oversupply of glycerol, essentially due to increasing biodiesel production, leads to decreasing prices and weak markets.

The price of pure glycerol varied from $0.50 to $1.50/lb and crude glycerol from $0.04/kg to $0.33/kg over the past few years. The price of glycerol in the market will continue to drop in such an over saturated market. Currently, the main supply of glycerol coming into the market is from the rapidly growing biodiesel industry. Estimated production of glycerol would reach 5.8 billion pounds in 2020. This is due to demand of biodiesel that is projected at 8 billion gallons in 2020 (Ayoub and Abdullah, 2012). Hence new outlets for glycerol are urgently needed, particularly in the case of crude glycerol released by the biodiesel processes. As glycerol is a nontoxic, edible, biodegradable compound, it will provide important environmental benefits to the new platform products.

In case of biosurfactant production, dramatically rising in biodegradable, non-toxic and eco-friendly alternative for chemical surfactants and the re-discovered opportunity of biosurfactants that gave rise to invention and investment ahead of the typical rigors of techno-economic modeling for the use of glycerol as a feed stock, leading typically to unmet expectations. Bacteria produce biosurfactants if grown on carbon sources such as glucose, glycerol, and various vegetable oils. Our research on biosurfactant production by bacteria indicates that glycerol can be used efficiently for biosurfactant production (Rahman et al., 2002).

The considerable interest in biosurfactants in the recent years is also due to their low toxicity, biodegradable nature and specificity, which would be very suitable to meet the European Surfactant Directive. Regulation EC No.: 648/2004 requires clear and precise description of the biodegradability of the surfactant and test methods to give assurance of its aerobic biodegradability. This regulation establishes rules designed to achieve the free movement of detergents and surfactants for detergents in the internal market while, at the same time, ensuring a high degree of protection of the environment and human health.

Surfactants constitute an important class of industrial chemicals and are widely used in almost every sector of modern industry. Most of the commercially available surfactants are chemical surfactants mainly, petroleum-derived. However, rapid advances in biotechnology and increased environmental awareness among consumers combined with expected new environmental legislation has provided further impetus for serious consideration of biological surfactants as possible alternatives to existing products.

## Biosurfactant's economic feasibility—what it takes to become a market leader?

As described in the previous section, there is enormous awareness among the consumers these days with regard to sustainability and global warming. The demand for bio-based technologies is ever increasing and “green solutions” are sought for every process. Rhamnolipids have promising properties and fulfill the eco-friendly criteria, one of the main drivers, but are still struggling to become market leaders. The economics of production is a major bottleneck in the outburst of commercialization of rhamnolipids and other biosurfactants (Table 2). There is still no downstream technology economical and convincing enough to recover and purify rhamnolipids at industrial scale. In case of biosurfactant production the downstream processing accounts for 70–80% of the entire production costs.

**Table 2:** **Cost of biosurfactant per liter of solution (diluted and the CMC based cost calculation carried out by Connolly et al., 2010)**.

**Biosurfactant**	**Origin**	**Supplier**	**ST mN/m**	**CMC (%)**	**Cost (£/L)**
BioFuture	Bacterial rhamnolipid	BioFuture Ltd. Dublin	28	0.08	0.02
Citrasolv	Orange peel	Cleveland Biotech Ltd., Teesside	30	0.9	0.01
EC601	Bacterial rhamnolipid	Ecochem Ltd., Canada	29	0.2	0.23
EC1800	Bacterial consortium	Ecochem Ltd., Canada	28	0.04	0.01
Petrosolv	Bacterial unknown	Enzyme Technologies Inc., USA	34	0.2	0.01
Saponin	Plant bark	Sigma UK	45	0.1	0.50

*The table also gives the origin of biosurfactant along with surface tension (ST) and critical micelle concentration (CMC) values*.

It is a no-brainer that in order to gain higher profit at commercial scale it requires access to very cheap feedstock. There are some other key parameters that need thorough consideration in order to make any product economically feasible. Technological fit and process optimization are among the main drivers. Fermentation time is another key to success. Fermentation performance and scale impact process economy as it is directly related to the yield, titer, and productivity. High cost of production especially because of the expensive substrates and down-stream processes makes it difficult to bring down the price of these environment friendly biomolecules. In order to compete with the synthetic detergents or surfactants the cost of production must be brought down to £1.70 per liter which is in itself a challenging task. As there are many barriers in the commercialization of biosurfactants, there seems no dearth of opportunities in this field. Cost comparison of various technologies viz. enzymatic, continuous, shake flask, batch, and fed-batch used for biosurfactant production pinpoint the requirement of innovative methods wherein rhamnolipids can be produced in static conditions to reduce the fermentation cost. The operating costs can be brought down by robust wild-type strains or recombinant mutant strains. Testing the possibility of co-products and/or enzymes is another attractive solution to surge the net profit—for example: esterases released during the production of lipopeptides by *Bacillus* strain and its recombinants (Sekhon et al., 2011, 2012). Co-products and by-products are value drivers and increase the economic viability of any business. The search of cheap and easily accessible raw material or substrate for biosurfactants production has been going on for years. The utilization of by-products, even if from a different process could be another smart solution—for example: glycerol, which is a by-product of biodiesel production, is available in surplus amount in the global market (Albarelli et al., 2011) which might be a cheap alternative for biosurfactant production.

Rhamnolipids are well-characterized and scientifically proven biosurfactants which are slowly and steadily becoming highly sought after biomolecules. Among other biosurfactants rhamnolipids have the highest number of patents (Table 3) and research publications. However, cost-competitiveness is one of the major factors that is holding rhamnolipids back from becoming the champions of their field. Research needs to be focused on suitable vigorous production strains, cheap substrates and nominal bioreactor technology. The current market price of rhamnolipid (R-95, 95%) is $227/10 mg (Sigma-aldrich) and $200/10 mg (AGAE technologies, USA) calling for strenuous research. Rhamnolipids have favorable applications in various sectors and if made economically sustainable nothing can stop these biomolecules to rule the surface-active compounds market.

**Table 3:** **A timeline and the major patents and grants obtained on rhamnolipids so far**.

**S. No**.	**Patent or Application No**.	**Filed**	**Issued**	**Title**	**Inventors**
1	4628030	Aug 1984	Dec 1986	Process for the production of rhamnolipids	Kaeppeli and Guerra-Santos
2	4814272	Feb 1985	March 1989	Process for the biotechnical production of rhamnolipids including rhamnolipids with only one. Beta.-hydroxydecanoic acid residue in the molecule	Wagner et al.
3	4933281	March 1987	June 1990	Method for producing rhamnose	Daniels et al.
4	4902512	Jan 1988	Feb 1990	Rhamnolipid liposomes	Ishigami et al.
5	5417879	Sep 1993	May 1995	Synergistic dual-surfactant detergent composition containing sophoroselipid	Hall et al.
6	5455232	April 1994	Oct 1995	Pharmaceutical preparation based on rhamnolipid	Piljac and Piljac
7	5550227	May 1994	Aug 1996	Method for the preparation of rhamnose monohydrate from rhamnolipids	Mixich et al.
8	5466675	July 1994	Nov 1995	Immunological activity of rhamnolipids	Piljac and Piljac
9	5520839	Jan 1995	May 1996	Laundry detergent composition containing synergistic combination of sophorose lipid and non-ionic surfactant	Hall et al.
10	5501966	Jan 1995	March 1996	*Pseudomonas aeruginosa* and its use in a process for the biotechnological preparation of L-rhamnose	Giani et al.
11	5658793	June 1995	Aug 1997	*Pseudomonas aeruginosa* and its use in a process for the biotechnological preparation of L-rhamnose	Giani et al.
12	5514661	Aug 1995	May 1996	Immunological activity of rhamnolipids	Piljac and Piljac
13	5767090	Jan 1996	June 1998	Microbially produced rhamnolipids (biosurfactants) for the control of plant pathogenic zoosporic fungi	Stanghellini et al.
14	7129218	Aug 2000	Oct 2006	Use of rhamnolipids in wound healing, treatment and prevention of gum disease and periodontal regeneration	Stipcevic et al.
15	7262171	Aug 2000	Aug 2007	Use of rhamnolipids in wound healing, treating burn shock, atherosclerosis, organ transplants, depression, schizophrenia and cosmetics	Piljac and Piljac
16	20040224905	May 2002	Nov 2004	Use of rhamnolipids in wound healing, treatment and prevention of gum disease and periodontal regeneration	Stipcevic et al.
17	20060233935	Nov 2003	Oct 2006	Rhamnolipids in bakery products	Haesendonck and Vanzeveren
18	7202063	Aug 2005	April 2007	Processes for the production of rhamnolipids	Gunther et al.
19	20070191292	Feb 2006	Aug 2007	Antimycotic rhamnolipid compositions and related methods of use	Gandhi et al.
20	20070155678	Feb 2007	July 2007	Use of rhamnolipids in wound healing, treating burn shock, atherosclerosis, organ transplants, depression, schizophrenia and cosmetics	Piljac and Piljac
21	20070207930	Feb 2007	Sep 2007	Rhamnolipid compositions and related methods of use	Gandhi et al.
22	7968499	Feb 2007	June 2011	Rhamnolipid compositions and related methods of use	Gandhi and Skebba
23	20080213194	July 2007	Sep 2008	Rhamnolipid-based formulations	Keith DeSanto
24	7985722	July 2007	July 2011	Rhamnolipid-based formulations	Keith DeSanto
25	20100249058	Oct 2007	Sep 2010	Feed additive and feed	Ito et al.
26	20090126948	Nov 2007	May 2009	Use of rhamnolipid based formulations for fire suppression and chemical and biological hazards	Keith DeSanto
27	20080261891	Feb 2008	Oct 2008	Compositions and methods for using syringopeptin 25A and rhamnolipids	Bart C. Weimer
28	20090220603	May 2009	Sep 2009	Use of rhamnolipids in wound healing, treating burn shock, atherosclerosis, organ transplants, depression, schizophrenia and cosmetics	Piljac and Piljac
29	20110123623	Nov 2010	May 2011	Rhamnolipid mechanism	Keith DeSanto
30	20120322751	Feb 2011	Dec 2012	Use of rhamnolipids as a drug of choice in the case of nuclear disasters in the treatment of the combination radiation injuries and illnesses in humans and animals	Goran Piljac
31	20110257115	June 2011	Oct 2011	Method for treating rhinitis and sinusitis by rhamnolipids	Anton Leighton
32	20110306569	June 2011	Dec 2011	Rhamnolipid biosurfactant from *Pseudomonas aeruginosa* strain NY3 and methods of use	Yin et al.
33	8592381	June 2011	Nov 2013	Method for treating rhinitis and sinusitis by rhamnolipids	Anton Leighton
34	20110270207	July 2011	Nov 2011	Rhamnolipid based formulations	Keith DeSanto
35	8183198	July 2011	May 2012	Rhamnolipid-based formulations	Keith DeSanto
36	20130130319	July 2011	May 2013	Cells and methods for producing rhamnolipids	Schaffer et al.
37	20120255918	April 2012	Oct 2012	Use of rhamnolipids in the water treatment industry	DeSanto and Keer
38	20130296461	May 2013	Nov 2013	Aqueous coatings and paints incorporating one or more antimicrobial biosurfactants and methods for using same	Lakshmi Sadasivan
39	20130310330	July 2013	Nov 2013	Method for treating obesity	Anton Leighton
40	8765694	July 2013	July 2014	Method for treating obesity	Anton Leighton
41	20140080771	Nov 2013	March 2014	Method for treating rhinitis and sinusitis by rhamnolipids	Anton Leighton
42	20140148588	Nov 2013	May 2014	Process for the isolation of rhamnolipids	Schilling et al.

## Concluding remarks

As the Health and Safety in the bioprocessing become paramount for large scale production there are significant interests in the search for novel non-pathogenic rhamnolipid producers. The numbers of cultured organisms from the environmental samples are only a tiny fraction (0.001%) of the actual microbial diversity. There are significant number of microbial isolates that needs to be explored and exploited for rhamnolipid and other bioproduct manufacturing. Biosurfactant producing probiotic organisms will play a key role in the future of biosurfactant market. Edible emulsifiers from these processes would be applicable to many applications including food, cosmetic, environmental clean-up, biomedical and natural therapy. Rhamnolipid could be a potential alternative for the synthetic surfactant molecules and an important platform chemical cluster with the market value of $2.8 billion in 2023 (Grand View Research Inc., 2014). There is a significant need for the discovery of novel non-pathogenic rhamnolipid producers with enhanced production capacity and efforts to scale up through bioprocess engineering are important to meet the future predictions of biosurfactants market.

### Conflict of interest statement

The authors declare that the research was conducted in the absence of any commercial or financial relationships that could be construed as a potential conflict of interest.
